# Evidence of an increased neuronal activation-to-resting glucose uptake ratio in the visual cortex of migraine patients: a study comparing ^18^FDG-PET and visual evoked potentials

**DOI:** 10.1186/s10194-018-0877-8

**Published:** 2018-07-05

**Authors:** Marco Lisicki, Kevin D’Ostilio, Gianluca Coppola, Felix Scholtes, Alain Maertens de Noordhout, Vincenzo Parisi, Jean Schoenen, Delphine Magis

**Affiliations:** 1 Headache Research Unit, University Department of Neurology CHR, CHU de Liège, Boulevard du 12eme de Ligne 1, 4000 Liege, Belgium; 2 G. B. Bietti Foundation IRCCS, Research Unit of Neurophysiology of Vision and Neuro-Ophthalmology, Rome, Italy; 3 Departments of Neurosurgery & Neuroanatomy, University of Liège, Liege, Belgium

**Keywords:** Headache, Cerebral energy metabolism, Astrocytes, Neurophysiology

## Abstract

**Background:**

Migraine attacks might be triggered by a disruption of cerebral homeostasis. During the interictal period migraine patients are characterized by abnormal sensory information processing, but this functional abnormality may not be sufficient to disrupt the physiological equilibrium of the cortex unless it is accompanied by additional pathological mechanisms, like a reduction in energetic reserves. The aim of this study was to compare resting cerebral glucose uptake (using positron emission tomography (^18^fluorodeoxyglucose-PET)), and visual cortex activation (using visual evoked potentials (VEP)), between episodic migraine without aura patients in the interictal period and healthy volunteers.

**Methods:**

Twenty episodic migraine without aura patients and twenty healthy volunteers were studied. ^18^FDG-PET and VEP recordings were performed on separate days. The overall glucose uptake in the visual cortex-to-VEP response ratio was calculated and compared between the groups. Additionally, PET scan comparisons adding area under the VEP curve as a covariate were performed. For case-wise analysis, eigenvalues from a specific region exhibiting significantly different FDG-PET signal in the visual cortex were extracted. Standardized glucose uptake values from this region and VEP values from each subject were then coupled and compared between the groups.

**Results:**

The mean area under the curve of VEP was greater in migraine patients compared to healthy controls. In the same line, patients had an increased neuronal activation-to-resting glucose uptake ratio in the visual cortex. Statistical parametric mapping analysis revealed that cortical FDG-PET signal in relation to VEP area under the curve was significantly reduced in migraineurs in a cluster extending throughout the left visual cortex, from Brodmann’s areas 19 and 18 to area 7. Within this region, case-wise analyses showed that a visual neuronal activation exceeding glucose uptake was present in 90% of migraine patients, but in only 15% of healthy volunteers.

**Conclusion:**

This study identifies an area of increased neuronal activation-to-resting glucose uptake ratio in the visual cortex of migraine patients between attacks. Such observation supports the concept that an activity-induced rupture of cerebral metabolic homeostasis may be a cornerstone of migraine pathophysiology.

This article has been selected as the winner of the 2018 Enrico Greppi Award. The Enrico Greppi Award is made to an unpublished paper dealing with clinical, epidemiological, genetic, pathophysiological or therapeutic aspects of headache. Italian Society for the Study of Headaches (SISC) sponsors this award, and the award is supported through an educational grant from Teva Neuroscience. This article did not undergo the standard peer review process for The Journal of Headache and Pain. The members of the 2018 Enrico Greppi Award Selection Committee were: Francesco Pierelli, Paolo Martelletti, Lyn Griffiths, Simona Sacco, Andreas Straube and Cenk Ayata.

## Background

Between attacks, migraine patients often exhibit abnormal sensory information processing [1]. Cortical hyper-responsivity during any kind of sensory stimulus repetition, including visual, is the most common electrophysiological feature found in episodic migraine patients during the interictal state [2]. Such alteration however, although highly prevalent among migraineurs, is by itself insufficient to entirely explain migraine’s pathophysiology [3]. The fact that similar cortical reactivity profiles can be found in both migraine patients and their asymptomatic first degree relatives suggests the existence of additional pathologic mechanisms in migraine sufferers that, when associated to visual hyper-responsivity, lead to the development of the disease [3].

In parallel with cortical hyper-responsivity, cerebral metabolism has been suggested to play a major role in migraine [4–6]. Hypotheses argue that reduced mitochondrial energy and ATP levels observed in the cortex of patients between attacks [7–9] might make them unable to deal with an energetically more demanding neuronal activity [5, 10]. This imbalance would later translate into a disruption in cortical homeostasis, with subsequent activation of the trigeminovascular system. Such view is supported by the favourable clinical response to metabolic enhancers [11] and ketogenic diet [12, 13] observed in migraine patients, as well as the capability of metabolic challenges to trigger migraine attacks [6].

^18^Fluorodeoxyglucose-positron emission tomography-(FDG-)PET is an imaging tool used to measure glucose uptake in brain tissue. FDG-studies in migraine patients between attacks have identified regions of reduced metabolism in limbic areas belonging to the pain/salience matrix [14, 15]. Yet, no interictal FDG-PET abnormalities affecting the visual cortex have been reported, with the possible exception of increased glucose uptake in posterior white matter [16] . Nonetheless, in available FDG-PET studies, no attempt was made to correlate cerebral metabolism with sensory processing.

In the present study we sought to determine if interictal glucose metabolism in the visual cortex is proportional to visually-induced neuronal activation. For this purpose, we assessed cerebral glucose uptake, and recorded visual evoked potentials (VEP) in healthy volunteers and migraine patients. Our main hypothesis was that the interictal responsivity of the visual cortex in migraine patients would exceed the resting glucose uptake, rendering migraineurs vulnerable to a disruption of metabolic homeostasis in times of increased neuroenergetic demands.

## Methods

### Study participants

The study involved twenty episodic migraine without aura patients (MO; mean age (SD): 31.1 (±12.6), 85% fem) diagnosed in accordance with The International Classification of Headache Disorders 3rd edition (Beta version) [17] and twenty healthy volunteers (HV; mean age (SD): 36.1 (±11.4), 75% fem) who did not report having first degree relatives suffering from recurrent headaches of any type. There were no significant differences in age or gender proportions between groups. Participants were recruited among University students or their families or via our headache clinic. None of them took any medication on a daily basis (other than the contraceptive pill), and they were all were free of any systemic or neurological disease other than migraine. Patients were not under any prophylactic treatment at the time of recordings, nor had they been for at least 30 preceding days. The mean number of monthly migraine days determined by headache diary inspection (during the month of the recordings) was 4.3 ± 2.5. All patients were recorded at an interval of at least 72 h before and after an attack. The study was approved by the Institution’s ethics committee (Centre Hospitalier Régional de la Citadelle, Liège, Belgium – protocol n°1422) and conducted following the principles of the Declaration of Helsinki. All participants gave their written informed consent.

### Visual evoked potentials recordings and analysis

Visual evoked potentials (VEP) were used as a marker of visual cortex responsiveness. Recordings were performed in the electrophysiology laboratory of the Headache Research Unit (Neurology Department, Centre Hospitalier Régional de la Citadelle, Liège, Belgium). Subjects sat on a comfortable armchair, in a quiet room with dimmed light. Needle electrodes were placed at Oz (active) and Fz (reference) of the 10–20 EEG system. With the left eye patched, participants were instructed to fixate on a red dot in the centre of a screen displaying a black and white reversing checkerboard pattern (contrast of 80%, mean luminance 50 cd/m2) at temporal and spatial stimulating frequencies of 1.55 Hz (3.1 reversals/second) and 68° respectively. Six hundred epochs, each lasting 250 ms, were uninterruptedly recorded at a sampling rate of 5.000 Hz using a CED™ power 1401 device (Cambridge Electronic Design Ltd., Cambridge, UK). After DC subtraction, recordings were exported to EEGLAB [18] (an open-source MATLAB (The MathWorks Inc.) toolbox for electrophysiological signal processing), where they were band-pass filtered (low pass 100 Hz, high pass 1 Hz). Artifacted epochs exceeding two standard deviations of the channel mean limit were rejected (< 7% of epochs). We extracted the area under the curve (AUC) of each single trial, and thereafter averaged these individual values. This method is deemed to be less affected by phase synchronization and thus to provide a more straightforward measure of neuronal activation [19].

### FDG-PET acquisition and analysis

PET acquisitions were made in the Radiodiagnostics Department of the Centre Hospitalier Universitaire (CHU) Sart Tilman, Liège, Belgium (Prof. R. Hustinx) using a Gemini TF PET/computed tomography (CT) scanner (Philips, Eindhoven, The Netherlands). Resting cerebral metabolism was studied 30 min after intravenous injection of 150 MBq FDG. Mean blood glucose level was 89.5 (range:110–77). Subjects were injected and scanned in a dark room with minimal environmental noise. They were instructed to maintain their eyes closed during the scan. Images were reconstructed using an iterative list mode time-of-flight algorithm. Corrections for attenuation, dead-time, random and scatter events were applied. PET acquisitions were analysed using Statistical Parametric Mapping version 12 (SPM12, Wellcome Trust Centre for Neuroimaging, http://www.fil.ion.ucl.ac.uk/spm) implemented in MATLAB 7.4.0 (MathWorks Inc., Sherborn, MA, USA). Images were first manually reoriented and hen spatially normalised into a standard stereotactic space using an MNI PET template (Montreal Neurological Institute) and smoothed using an 8 mm full-width-half-maximum (FWHM) isotropic kernel. We performed global normalisation by applying proportional scaling. Cerebral regions identification and masks were established using the WFU PickAtlas toolbox (Wake Forest University School of Medicine, Advanced NeuroScience Imaging Research lab (ANSIR), Winston-Salem, NC, U.S.A). PET scans were compared between groups (1) without adding any covariates, and (2) using the interaction with the averaged area under the VEP as regressor. An exploratory (*p* < 0.001 uncorrected) analysis followed by a more stringent comparison (p family-wise error[FWE] < 0.05 correction at a whole brain level) were performed. Only statistically significant clusters within regions of the visual cortex are reported in the results. To estimate the neuronal activation-to-overall resting glucose uptake ratio in the visual cortex, eigenvalues corresponding to Brodmann’s areas 17, 18, and 19 were extracted using a volumetric mask. The ratio between these values, and the mean area under the VEP curve was then calculated. In addition, based on the statistical parametric mapping results, for case-wise analysis a specific volumetric mask was generated upon the cluster in the visual cortex exhibiting a statistically significant difference in FDG-uptake between groups when including VEP-AUC as a covariate. By using this volumetric mask, eigenvalues corresponding to this specific region were extracted for each subject.

### Statistical analysis

Statistical analyses were performed in Prism version 6.00 for Windows (GraphPad Software, La Jolla, California, USA). Continuous variables were compared using the t-test. The assumption of normality was evaluated using a Shapiro-Wilk normality test. For case-wise analyses, mean area under the VEP curve (accounting for visual activity) and eigenvalues corresponding to the specific cluster of the visual cortex exhibiting significant FDG-uptake differences (accounting for metabolic activity) were Z-transformed and paired for each subject. Proportions of participants with a visual > metabolic Z-score in each group were compared using Fisher’s exact test. The significance level was set at *p* < 0.05 for all statistical analyses.

## Results

**Fig. 1:**
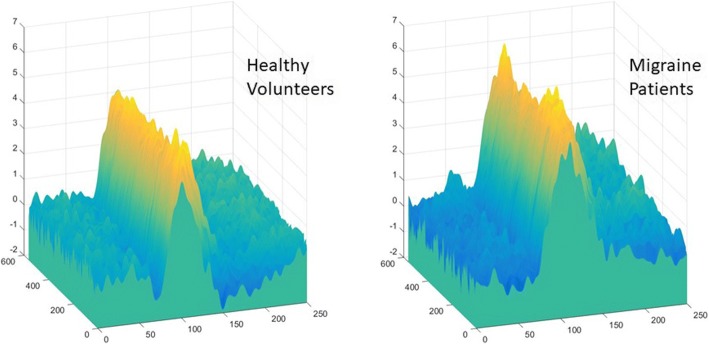
Mean Visual Evoked potentials (x = time in ms, y = trial number, z = amplitude in μV) in healthy volunteers (left) and episodic migraine patients (right). The area under the curve was calculated with respect to the baseline (zero)

The mean area under the curve of VEP was significantly greater in migraine patients compared to healthy controls (1091 μV^2^ ± 164.8 vs. 362.8 μV^2^ ± 167.7; *p* < 0.01) (Fig. 1).

Four clusters of reduced metabolism where initially observed (uncorrected *p* < 0.001 level) in the visual cortex of migraine patients, but they did not withstand the correction for multiple comparisons. No clusters of increased PET signal were found in the visual cortex of migraineurs.

The mean ratio between the VEP-AUC and overall glucose uptake in the visual cortex (BA 17, 18 and 19) was three times higher in migraine patients (12,73 ± 1947 vs. 4.183 ± 1955; p < 0.01).

**Fig. 2:**
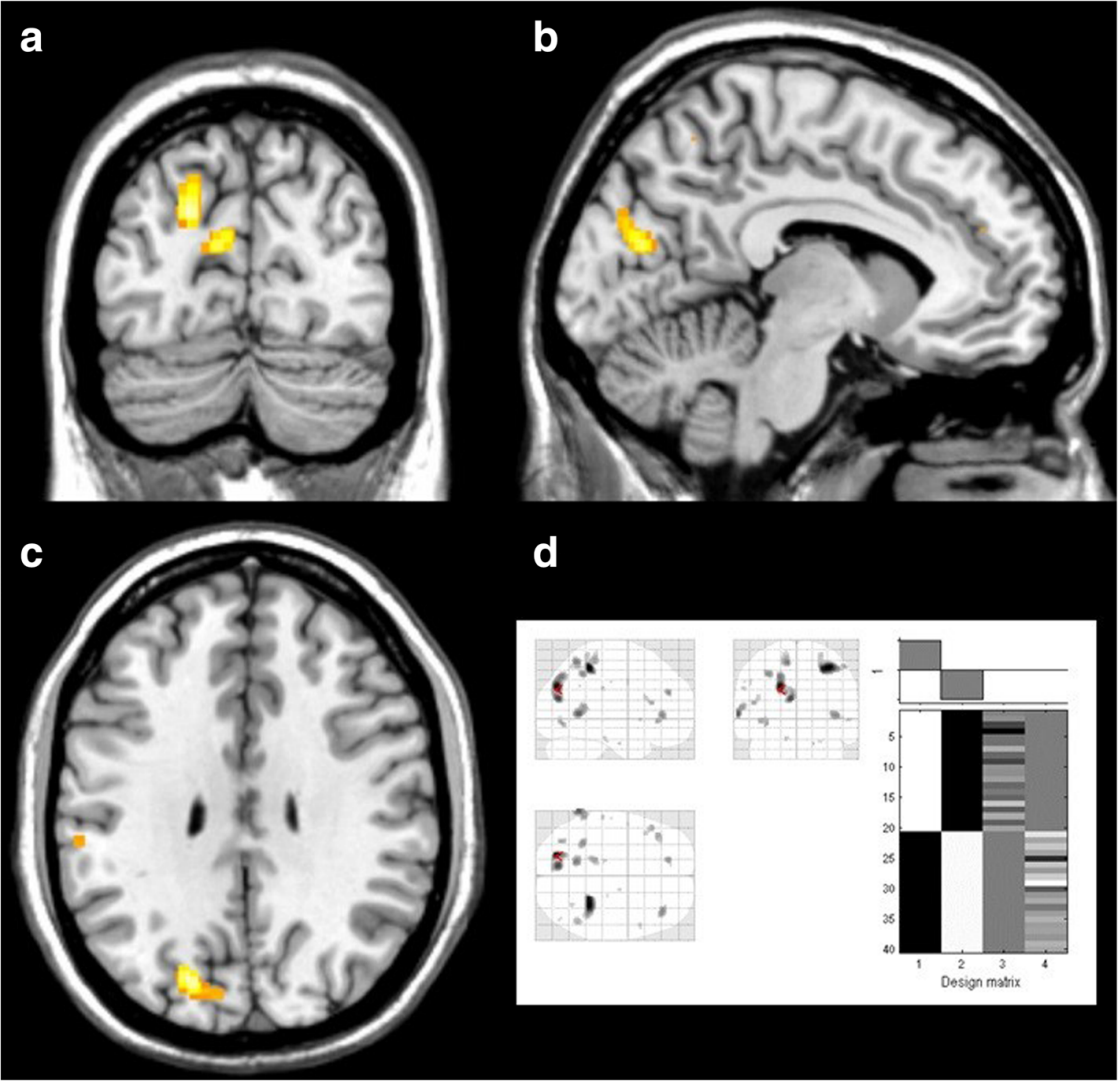
Areas of increased metabolism in healthy controls compared with migraine patients using the interaction with area under the VEP as regressor. Clockwise from top left: (**a**) coronal view, (**b**) sagittal view, (**c**) axial view, and (**d**) “glass brain” representation and design matrix

**Fig. 3:**
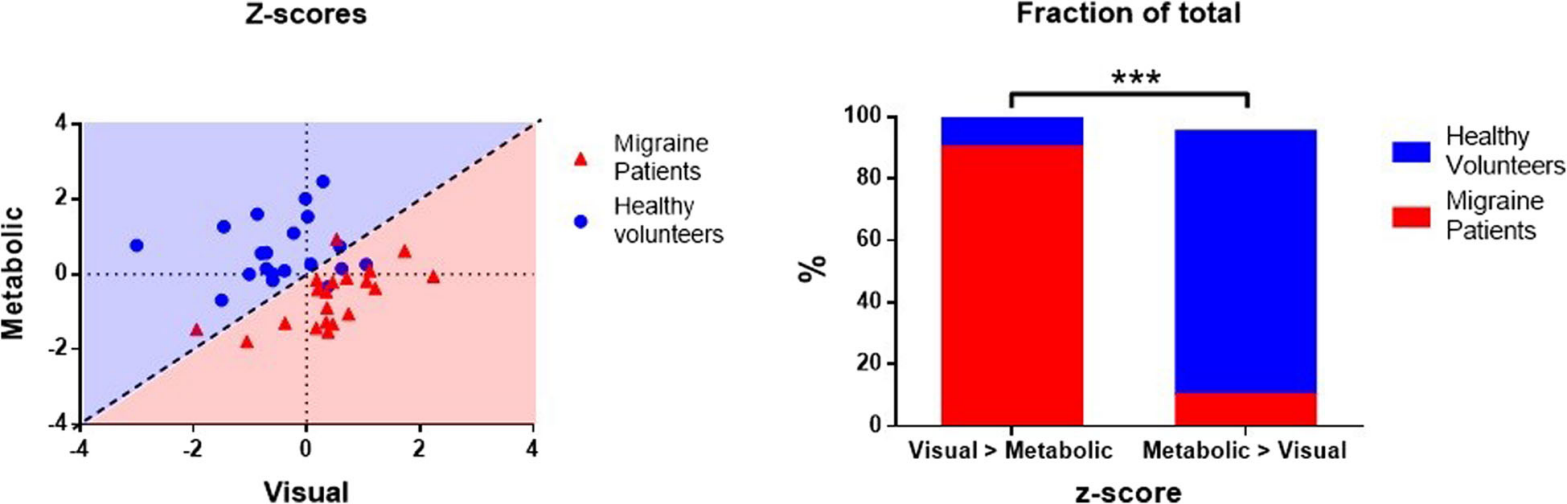
Left: Metabolic and Visual Z-scores of each participant plotted on a Cartesian coordinates plane. Points above the dashed diagonal line (light blue shaded area) correspond to participants with higher glucose uptake in the visual cortex with respect to their visual responsiveness score. Points below the dashed diagonal line (light red shaded area) correspond to participants exhibiting disproportionally higher visual responses considering their glucose uptake. Right: relative proportion of subjects of each within each side of the dashed diagonal line of the Cartesian plot. The asterisks (***) indicate a *p* value < 0.001

When adding VEP-AUC as a covariate, statistical parametric mapping comparisons revealed a cluster of significantly reduced uptake in patients, with three peaks extending over Brodmann’s areas 19, 18 and 7 on the left (− 20, − 78, 30 *T* = 5.66 peak-level p [FWE-corr] = 0.039; − 8, − 78, 20 *T* = 4.84; − 20, − 74, 42 *T* = 4.36, all cluster-level pFWE-corr = 0.021) (Fig. 2). Eigenvalues from this specific cluster were extracted and standardized for case-wise analysis, where a visual activation Z-score exceeding the glucose uptake Z-score was found in 90% of migraine patients, but in only 15% of healthy volunteers (*p* < 0.001, Fisher’s exact test) (Fig. 3).

## Discussion

In this study, using ^18^fluorodeoxyglucose-positron emission tomography acquired at rest and pattern reversal-visual evoked potentials, we compared interictal glucose uptake and neuronal activation in the visual cortex of migraine patients and healthy volunteers. Our FDG-PET data confirm the finding of an interictally decreased metabolism in the occipital areas previously shown using phosphorus magnetic resonance spectroscopy [7–9]. An additional, novel finding, is that the ratio between stimulation-induced neuronal activation and resting glucose uptake differs between migraine patients and healthy controls. Using statistical parametric mapping, we identified the specific region of the visual cortex where this difference reaches its maximum. The potential pathophysiological implications of these findings are discussed below.

According to most neurophysiological studies, migraine patients exhibit cortical hyper-responsivity to repeated sensory stimuli between attacks [20]. Although this may constitute a favourable evolutionary trait [21], enhanced responsivity has a price to be paid in terms of cerebral metabolism. In the brain, the visual system ranks amongst the most energy-consuming systems [22]. This renders it more susceptible to metabolic imbalance when energetic demands increase, energetic supplies decrease, or in a combination of both.

The occurrence of a metabolic strain in the cortex is a plausible hypothesis for igniting a migraine attack [23]. The cornerstone of the metabolic strain model is the unbalanced relationship between enhanced sensory processing and reduced metabolic offers [5]. According to this model, unmet neuronal metabolic demands in the cortex would result in a focal disruption of homeostasis and release of molecules capable of activating the trigemino-vascular system [24–26]. This mechanism, although orchestrated by neurons [26], relies on astrocytes as concertmasters [27]. By determining an area of sensory-metabolic mismatch (suggestive of the presence of a higher cortical responsivity and relatively lower resting energy reserves) in the visual cortex of migraine patients, our results provide supportive experimental evidence to the initiatory phase of the metabolic strain model. Intuitively, energy reserves may be further decreased by daily life stressful and energy consuming events [28], or by the sensory hypersensitivity that patients present even between crises, which extends up to the premonitory phase [29] when, along with the energetic deficit, may worsen up to the tipping point of the attack. This view is supported by a previous study in which the authors simultaneously recorded VEP and cerebral blood flow velocity responses (VEFR) in a group of migraineurs. They observed an interictal increase of neurovascular coupling in patients interictally that, empirically, corresponds to higher neural activity, which might further reduce energy reserves [30]. Hence, the presence of an increase of energetic requirements in the absence of adequate energetic supplies would be a plausible reason for the brain to activate its major alarm system, the trigeminovascular system, that might ignite a migraine attack in order to prevent the system from overloading, and thus re-establish cortical homeostasis [4]. This is supported by three out of four studies where authors positively triggered migraine using sensory overload [31–34]. On the other hand, metabolic challenges have also been shown to provoke migraine attacks. From an observational perspective, skipping meals is the third most common migraine trigger reported by patients [35]. In the laboratory, Schoonman et al. reported provoking headache in six out of fourteen migraine patients exposed to normobaric hypoxia [36], and more recently, in an elegantly designed study also adressing the effects of hypoxia, Arngrim et al. reported migraine-like attacks in eight out of fifteen migraine patients, accompanied by definite aura in three, and possible aura in another four [37].

Our results do not necessarily pull in one direction, favouring sensory over metabolic factors or vice versa, but they rather imply that the physiological sensory-metabolic equilibrium is abnormal in migraine between attacks. However, it is of interest that resting brain energy metabolism remains low even during migraine attacks [38–40] when sensory hyper-responsivity tends to normalize [29]. This suggests that the former might be a trait factor permanently predisposing to the next crisis, while the latter would be more of a state-dependent phenomenon.

As mentioned before, the sensory profile of migraine patients makes them susceptible of sensory overload [1], and therefore in the need of protective mechanisms. Compensatory metaplastic changes have been hypothesized to explain the between-subject variability of electrophysiological responses in migraine groups in previous studies [41]. Thus, it could be debated whether the reduced resting glucose uptake in migraine patients we observed constitutes compensatory mechanism aiming to decrease cortical activation. However, given the heightened visual responsiveness we recorded in patients, this alternative hypothesis seems unlikely.

**Fig. 4:**
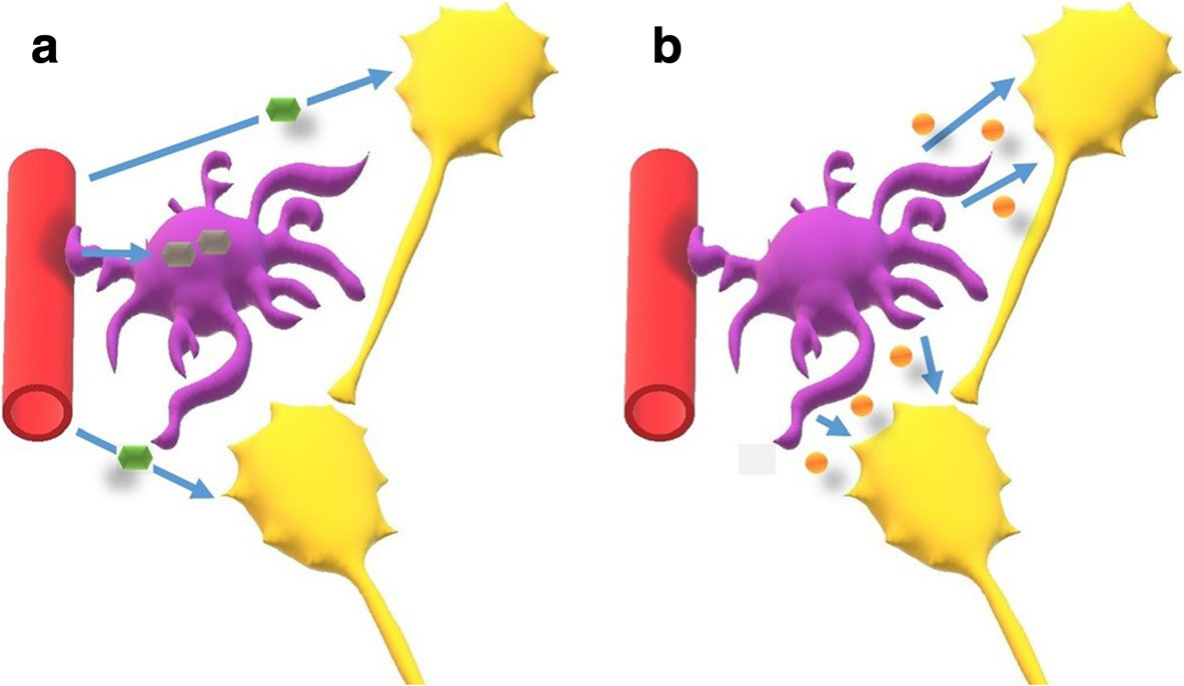
Schematic representation of astrocytic (purple) and neuronal (yellow) metabolism at rest (**a**, left) and upon stimulation (**b**, right). Only astrocytes accumulate glucose (green) at rest, whereas upon stimulation, energetic reserves are degraded in order to provide energy substrates (lactate, orange) to neurons. Astrocytes in exchange reuptake exceeding glutamate at the synaptic cleft level (not shown). For details see ref. [42]

One cannot discuss cortical metabolism and homeostasis without considering the role of astrocytes (a deficient uptake of glucose by astrocytes could explain our FDG-PET findings in migraine patients based on current evidence [42–44]). Neurons have a very scarce, if any, energetic reserve. Because of that, their increased metabolic demands upon stimulation basically rely on the energetic supplies provided by astrocytes. Unlike neurons, during rest astrocytes store glucose in the form of glycogen. When the cortex is stimulated, glycogen reserves in astrocytes are rapidly transformed into less complex energetic substrates, which are then provided to neurons in order to fulfil their enhanced metabolic needs (Fig. 4) [42]. In exchange, astrocytes buffer excessive glutamate molecules released in the synaptic cleft [45]. Other functions of astrocytes, besides regulating neuro-metabolic coupling and glutamate concentrations, include controlling the amount of extracellular potassium [46], and acting as regulators of cerebral blood flow [47]. Indeed, the influence of astrocytes on synaptic transmission is such, that the concept of a tripartite synapse (presynaptic neuron, astrocyte, and postsynaptic neuron) is steadily solidifying [48]. In fact, when altered, deficient clearance of potassium and glutamate by astrocytes seems to explain the pathogenesis of specific forms of migraine with monogenetic inheritance [49]. For the case of more common types of migraine, the pathophysiological implication of astrocytes is less well elucidated, although genetic studies seem to also point in their direction [50, 51].

Interestingly, the visual cortex has the lowest astrocyte/neuron density in the brain, which renders it more susceptible to homeostatic disturbances [52, 53]. Prior clinical [54] and experimental [55] evidence has suggested that waves of cortical spreading depression, the culprit of migraine aura, originate in the visual cortex. Visual area V3A was specifically pointed to as the initiating region in one report [53]. Another study described increased cortical thickness of this area in migraine without aura patients [56]. It might be worthwhile to mention that the cluster of mismatch we found in the present study extends over visual areas V3A and V7. Whether this observation is coincidental or entails clinical significance remains to be determined.

Our study has several limitations. First, PET scans and VEP where not recorded concomitantly and thus, the possibility of an important compensatory metabolic increase in migraine patients during stimulation remains open. Also, it is worth mentioning that a cycling pattern of increased susceptibility probably determined by the hypothalamus, or its limbic controls, is probably involved in migraine pathogenesis [57], and thus sensory-metabolic coupling might be preserved at one time point and impaired at another. Finally, even if there exists a metabolic imbalance during stimulation in the visual cortex of migraine patients, secondary activation of the trigeminovascular system would need to be further ascertained. In the future, studies addressing these issues would be necessary to clarify the possible role of a disruption in cortical homeostasis due to a sensory-metabolic disequilibrium in migraine pathophysiology.

## Conclusion

Our findings indicate the presence of an area of increased neuronal activation-to-resting glucose uptake ratio in the visual cortex of migraine patients between attacks. Despite some methodological reservation, this observation supports the concept that an activity-induced rupture of cerebral metabolic homeostasis may be a cornerstone in migraine pathophysiology. The potential physiopathological implications of such finding should be explored in depth in future studies.

## Abbreviations

^18^FDG-PET: ^18^fluorodeoxyglucose-positron emission tomography
AUC: Area under the curve
VEP: Visual evoked potentials

## References

[1] Goadsby PJ, Holland PR, Martins-Oliveira M (2017). Pathophysiology of migraine: a disorder of sensory processing. Physiol Rev.

[2] Magis D, Vigano A, Sava S (2013). Pearls and pitfalls: electrophysiology for primary headaches. Cephalalgia.

[3] Lisicki M, Ruiz-Romagnoli E, D’Ostilio K, et al (2017) Familial history of migraine influences habituation of visual evoked potentials. Cephalalgia 37. 10.1177/0333102416673207 10.1177/0333102416673207 27821640

[4] Schoenen J (1996). Deficient habituation of evoked cortical potentials in migraine: a link between brain biology, behavior and trigeminovascular activation?. Biomed Pharmacother.

[5] Schoenen J (1994). Pathogenesis of migraine: the biobehavioural and hypoxia theories reconciled. Acta Neurol Belg.

[6] Schoenen J (2016). Hypoxia, a turning point in migraine pathogenesis?. Brain.

[7] Reyngoudt H, Paemeleire K, Descamps B (2011). 31P-MRS demonstrates a reduction in high-energy phosphates in the occipital lobe of migraine without aura patients. Cephalalgia.

[8] Montagna P, Cortelli P, Monari L (1994). 31P-magnetic resonance spectroscopy in migraine without aura. Neurology.

[9] Lodi R, Montagna P, Soriani S (1997). Deficit of brain and skeletal muscle bioenergetics and low brain magnesium in juvenile migraine: an in vivo 31P magnetic resonance spectroscopy Interictal study. Pediatr Res.

[10] Gantenbein AR, Sandor PS, Fritschy J (2013). Sensory information processing may be neuroenergetically more demanding in migraine patients. Neuroreport.

[11] Schoenen J, Jacquy J, Lenaerts M (1998). Effectiveness of high-dose riboflavin in migraine prophylaxis. A randomized controlled trial. Neurology.

[12] Di Lorenzo C, Coppola G, Sirianni G (2015). Migraine improvement during short lasting ketogenesis: a proof-of-concept study. Eur J Neurol.

[13] Strahlman RS (2006). Can ketosis help migraine sufferers? A case report. Headache.

[14] Kim JH, Kim S, Suh SI (2010). Interictal metabolic changes in episodic migraine: a voxel-based FDG-PET study. Cephalalgia.

[15] Magis D, D’Ostilio K, Thibaut A (2017). Cerebral metabolism before and after external trigeminal nerve stimulation in episodic migraine. Cephalalgia.

[16] Kassab M, Bakhtar O, Wack D, Bednarczyk E (2009). Resting brain glucose uptake in headache-free migraineurs. Headache.

[17] Headache Classification Committee of the International Headache Society (IHS) (2013). The international classification of headache disorders, 3rd edition (beta version). Cephalalgia.

[18] Delorme A, Makeig S (2004). EEGLAB: an open source toolbox for analysis of single-trial EEG dynamics including independent component analysis. J Neurosci Methods.

[19] Makeig S, Debener S, Onton J, Delorme A (2004). Mining event-related brain dynamics. Trends Cogn Sci.

[20] de Tommaso M, Ambrosini A, Brighina F (2014). Altered processing of sensory stimuli in patients with migraine. Nat Rev Neurol.

[21] Loder E (2002). What is the evolutionary advantage of migraine?. Cephalalgia.

[22] Wong-Riley M (2010) Energy metabolism of the visual system. Eye Brain:99. 10.2147/EB.S9078 10.2147/EB.S9078 PMC3515641 23226947

[23] Paemeleire K, Schoenen J (2013). (31) P-MRS in migraine: fallen through the cracks. Headache.

[24] Zhang X, Levy D, Kainz V (2011). Activation of central trigeminovascular neurons by cortical spreading depression. Ann Neurol.

[25] Kilic K, Karatas H, Dönmez-Demir B (2018). Inadequate brain glycogen or sleep increases spreading depression susceptibility. Ann Neurol.

[26] Karatas H, Erdener SE, Gursoy-Ozdemir Y (2013). Spreading depression triggers headache by activating neuronal Panx1 channels. Science.

[27] Pietrobon D, Moskowitz MA (2014). Chaos and commotion in the wake of cortical spreading depression and spreading depolarizations. Nat Rev Neurosci.

[28] Lisicki M, Ruiz-Romagnoli E, Piedrabuena R, et al (2017) Migraine triggers and habituation of visual evoked potentials. Cephalalgia 333102417720217. 10.1177/0333102417720217 10.1177/0333102417720217 28691517

[29] Judit A, Sándor PS, Schoenen J (2000). Habituation of visual and intensity dependence of auditory evoked cortical potentials tends to normalize just before and during the migraine attack. Cephalalgia.

[30] Zaletel M, Strucl M, Bajrovic FF, Pogacnik T (2005). Coupling between visual evoked cerebral blood flow velocity responses and visual evoked potentials in migraneurs. Cephalalgia.

[31] Hougaard A, Amin F, Hauge AW (2013). Provocation of migraine with aura using natural trigger factors. Neurology.

[32] Martin PR, Seneviratne HM (1997). Effects of food deprivation and a stressor on head pain. Health Psychol.

[33] Cao Y, Aurora SK, Nagesh V (2002). Functional MRI-BOLD of brainstem structures during visually triggered migraine. Neurology.

[34] Cao Y, Welch KM, Aurora S, Vikingstad EM (1999). Functional MRI-BOLD of visually triggered headache in patients with migraine. Arch Neurol.

[35] Kelman L (2007). The triggers or precipitants of the acute migraine attack. Cephalalgia.

[36] Schoonman G, Sándor P, Agosti R (2006). Normobaric hypoxia and nitroglycerin as trigger factors for migraine. Cephalalgia.

[37] Arngrim N, Schytz HW, Britze J (2016). Migraine induced by hypoxia: an MRI spectroscopy and angiography study. Brain.

[38] Welch KM, Levine SR, D’Andrea G (1989). Preliminary observations on brain energy metabolism in migraine studied by in vivo phosphorus 31 NMR spectroscopy. Neurology.

[39] Welch KM, Levine SR, D’Andrea G, Helpern JA (1988). Brain pH in migraine: an in vivo phosphorus-31 magnetic resonance spectroscopy study. Cephalalgia.

[40] Ramadan NM, Halvorson H, Vande-Linde A (1989). Low brain magnesium in migraine. Headache.

[41] Cosentino G, Fierro B, Vigneri S (2014). Cyclical changes of cortical excitability and metaplasticity in migraine: evidence from a repetitive transcranial magnetic stimulation study. Pain.

[42] Bélanger M, Allaman I, Magistretti PJ (2011). Brain energy metabolism: focus on astrocyte-neuron metabolic cooperation. Cell Metab.

[43] Zimmer ER, Parent MJ, Souza DG (2017). [18F]FDG PET signal is driven by astroglial glutamate transport. Nat Neurosci.

[44] Figley CR, Stroman PW (2011). The role(s) of astrocytes and astrocyte activity in neurometabolism, neurovascular coupling, and the production of functional neuroimaging signals. Eur J Neurosci.

[45] Anderson CM, Swanson RA (2000). Astrocyte glutamate transport: review of properties, regulation, and physiological functions. Glia.

[46] Kimelberg HK, Nedergaard M (2010). Functions of astrocytes and their potential as therapeutic targets. Neurotherapeutics.

[47] Gordon GRJ, Choi HB, Rungta RL (2008). Brain metabolism dictates the polarity of astrocyte control over arterioles. Nature.

[48] Araque A, Parpura V, Sanzgiri RP, Haydon PG (1999). Tripartite synapses: glia, the unacknowledged partner. Trends Neurosci.

[49] Capuani C, Melone M, Tottene A, et al (2016) Defective glutamate and K+ clearance by cortical astrocytes in familial hemiplegic migraine type 2. EMBO Mol Med. 10.15252/emmm.201505944 10.15252/emmm.201505944 PMC4967947 27354390

[50] Eising E, De Leeuw C, Min JL, et al Involvement of astrocyte and oligodendrocyte gene sets in migraine. 10.1177/0333102415618614 10.1177/0333102415618614 26646788

[51] Renthal W Localization of migraine susceptibility genes in human brain by single-cell RNA sequencing. 10.1177/0333102418762476 10.1177/0333102418762476 29498289

[52] Lauritzen M, Dreier JP, Fabricius M (2011). Clinical relevance of cortical spreading depression in neurological disorders: migraine, malignant stroke, subarachnoid and intracranial hemorrhage, and traumatic brain injury. J Cereb Blood Flow Metab.

[53] Hadjikhani N, Sanchez Del Rio M, Wu O (2001). Mechanisms of migraine aura revealed by functional MRI in human visual cortex. Proc Natl Acad Sci U S A.

[54] Lashley KS (1941). Patterns of cerebral integration indicated by the scotomas of migraine. Arch Neurol Psychiatr.

[55] Lauritzen M, Olesen J (1984). Regional cerebral blood flow during migraine attacks by Xenon-133 inhalation and emission tomography. Brain.

[56] Granziera C, DaSilva AFM, Snyder J (2006). Anatomical alterations of the visual motion processing network in migraine with and without Aura. PLoS Med.

[57] Schulte LH, May A (2016). The migraine generator revisited: continuous scanning of the migraine cycle over 30 days and three spontaneous attacks. Brain.

